# Microbiological impact of long-term wine grape cultivation on soil organic carbon in desert ecosystems: a study on rhizosphere and bulk sandy soils

**DOI:** 10.3389/fpls.2024.1362149

**Published:** 2024-03-07

**Authors:** Zhiheng Wang, Wenchao Li, Yuejuan Wang, Xuefei Wang, Tingting Ma, Yanlin Liu, Yuqing Wei

**Affiliations:** ^1^ College of Biological Science & Engineering, North Minzu University, Yinchuan, Ningxia, China; ^2^ Administrative Committee of Wine Industry Zone of Ningxia Helan Mountains’ East Foothill, Yinchuan, Ningxia, China; ^3^ College of Enology, Northwest A&F University, Yangling, Shaanxi, China

**Keywords:** soil organic carbon, microbiological mechanism, grapevine, desert ecosystems, rhizosphere.

## Abstract

The improvement of nutrients in soil is essential for using deserts and decertified ecosystems and promoting sustainable agriculture. Grapevines are suitable crops for desert soils as they can adapt to harsh environments and effectively impact soil nutrients; however, the mechanisms underlying this remain unclear. This study explored the impact of the different duration(3, 6, and 10 years) of grape cultivation on soil organic carbon, physicochemical properties, enzyme activities, microbial communities, and carbon cycle pathways in both rhizosphere and bulk soils. Partial least squares path modeling was used to further reveal how these factors contributed to soil nutrient improvement. Our findings indicate that after long-term grape cultivation six years, soil organic carbon, total nitrogen, total phosphorus, microbial biomass carbon and nitrogen, and enzyme activities has significantly increased in both rhizosphere and bulk soils but microbial diversity decreased in bulk soil. According to the microbial community assembly analysis, we found that stochastic processes, particularly homogenizing dispersal, were dominant in both soils. Bacteria are more sensitive to environmental changes than fungi. In the bulk soil, long-term grape cultivation leads to a reduction in ecological niches and an increase in salinity, resulting in a decrease in soil microbial diversity. Soil enzymes play an important role in increasing soil organic matter in bulk soil by decomposing plant litters, while fungi play an important role in increasing soil organic matter in the rhizosphere, possibly by decomposing fine roots and producing mycelia. Our findings enhance understanding of the mechanisms of soil organic carbon improvement under long-term grape cultivation and suggest that grapes are suitable crops for restoring desert ecosystems.

## 1 Introduction

Approximately 40% of the global area is arid and semi-arid desert land, with vegetation mainly comprising small xerophytic trees, shrubs, and seasonal saltworts and other herbaceous plants (Maggi et al., 2018). Desert ecosystems are exposed to high evaporation, scarce precipitation, and large diurnal temperature differences over long periods, resulting in low vegetation cover and soil exposure (Vásquez-Dean et al., 2020). Desert ecosystems account for 44% of China’s total land area and are mainly distributed in 12 provinces, including Xinjiang, Gansu, and Ningxia (Wang et al., 2022b). Currently, xeric shrub, sandy, gravel, saline-alkaline, clay-based, and alpine-cold desert ecosystems are found in China, accounting for 6.34%, 27.49%, 26.99%, 6.88%, 19.98%, and 12.31% of the total desert ecosystem area, respectively (Wang et al., 2022b). Sandy soils, with an average sand content >50% and a clay content of <20% up to a depth of 30 cm, are widely distributed worldwide (Huang and Hartemink, 2020). These sandy soils have low nutrient contents, particularly soil organic matter (SOM). In the desert grasslands of Ningxia and other places in China, the average SOM content in sandy soil is approximately 7 g kg^-1^ (Gao et al., 2014). Low soil fertility is a major factor limiting development in these areas.

Desert ecosystems are characterized by water scarcity, high surface evaporation, and harsh climates, which make vegetation survival difficult and limit plant-derived carbon sources (Throop et al., 2013). Desert ecosystems, where soil carbon storage is mainly organic through the action of fungi or other soil microorganisms, have gradually developed a different soil carbon cycle pathway than other ecosystems that rely on plant litter, a carbon cycle mode with the coexistence of organic and inorganic carbon (Wang et al., 2010; An et al., 2019; Du et al., 2021). Vegetation restoration in decertified land is one of the main strategies for regulating water and soil erosion, increasing soil carbon content, and restoring ecosystems (Deng et al., 2014; Ding et al., 2022). Grapes have a high nutritional value, are adaptable, and are cultivated worldwide, often in arid or semi-arid climates (Koundouras et al., 2008). In Ningxia, northwest China, grapes are planted as economic crops. Researchers have found that long-term planting of wine grapes significantly increases vegetation cover and SOM content, decreases blowing sand, and increases the total annual value of ecosystem services (Capó-Bauçà et al., 2019; Zhang et al., 2021). Belmonte et al. (2018) reported that long-term grape cultivation (22 years) increased soil carbon input under no-tillage. Moreover, long-term grape cultivation not only improved soil organic carbon (SOC) content in the topsoil, but also significantly increased SOC content in the subsoil (Jakšić et al., 2021).Therefore, revealing the mechanism of long-term grape cultivation on sandy soil improvement plays an important role in desert ecosystem restoration.

SOC primarily originates from plant photosynthesis, fixed CO_2_, and exogenous carbon inputs, such as fertilizer (Wei et al., 2021). After receiving the organic carbon, the soil microbial community continuously drives soil nutrient conversion through microbial carbon cycling and metabolic pathways, promoting the formation of recalcitrant organic carbon (Fu et al., 2021). Owing to the various organic matter components in the rhizosphere and bulk soils, different soil microbial functional communities were formed in these two regions (Nuccio et al., 2020). Recent studies have found that the fungal necromass content around the rhizosphere of arbuscular mycorrhizal tree species is lower than that in bulk soil, and that saprotrophic bacteria inhibit the decomposition of recalcitrant organic carbon (Jia et al., 2023). However, some studies have also shown that easily decomposable organic carbon secreted by roots promotes the microbial decomposition of SOM (Chen et al., 2014; Huo et al., 2017). In bulk soil, microorganisms decompose plant residues, such as litter and straw, or use nutrients in the soil fertilizer to synthesize their own components and cell exudates by aggregating and binding with other particles in the soil (Uhlířová et al., 2005; Miltner et al., 2012). There is no natural boundary between the rhizosphere and bulk soils; therefore, organic carbon sources in the soils can transfer and diffuse with each other. Plant roots can also alter the organic carbon composition and content of bulk soil via microbial gradient cooperation (Sun et al., 2023).

However, only a limited number of studies have offered a comprehensive account of the alterations in microbial communities and the mechanisms of nutrient transport between the rhizosphere (the soil surrounding plant roots) and bulk soils. These changes are primarily mediated by microorganisms in soils that have been used for long-term grape cultivation. This study measured the changes in physicochemical properties and microbial community structure of rhizosphere and bulk soils in soils without grape cultivation in vineyards in the eastern Helan Mountain, Ningxia. After 3, 6, and 10 years of grape cultivation, the improvement effect on desert sandy soil was measured, and the transfer and transformation mechanism of samples between rhizosphere and bulk soils mediated by microorganisms based on the microbial community and functional structure was assessed.

## 2 Materials and methods

### 2.1 Study area

The sampling site (Ningxia Zhihuiyuanshi Winery, (28.579° N, 106.041° E) is located at an altitude of 1,240–1,450 m in the eastern Helan Mountains, Ningxia Province, China (**Supplementary Figure S1**). As a nationally protected area, the sampling site has a desert climate with an average annual precipitation of 251 mm. The main soil type at the sampling site is sandy soil (50% clay, 30% silt, 20% sand). *Populus L.*, *Zygophyllaceae*, *Ulmusglaucescens*, *Leguminosae*, and *Elaeagnaceae* are plants found in the sampling area. For 10 years, a recovery experiment with long-term planting was conducted. All vineyards were well managed, with plants arranged in north–south rows (row spacing of 3 m, vine spacing of 0.5 m) and the single cane “Cabernet Sauvignon” cultivation method. Fertilizer was applied once a year in the field in November when the grapes underwent burial. Irrigation was done by drip irrigation with 150 m^3^/ha each time during budding, flowering, fruit set, color change and pre-harvest.

### 2.2 Experimental design and sample

The sampling area was divided into four regions, each corresponding to grape cultivation initiated 3, 6, and 10 years ago, as well as a region where no grapes were planted (**Supplementary Figure S2**). In August 2022, a five-point sampling method was employed to determine the soil sampling experimental plots from the 3-, 6-, and 10-year restoration periods, as well as the region where no grapes were planted. In each experimental plot, three soil samples were randomly collected at depths of 0–30 cm. These samples were combined into a single sample, a process that was repeated five times during the 3-, 6-, and 10-year restoration periods. The soil that adhered to the roots after shaking was defined as rhizosphere soil, whereas the remaining soil was homogeneously mixed and defined as bulk soil. A desert without grape cultivation was used as the control. All soil samples were sieved (mesh width of 2 mm) to remove impurities before further processing. Forty soil samples were obtained in total. Each soil sample was divided: one part was air-dried for pH, total carbon, total nitrogen, total phosphorus, total potassium, organic matter, available phosphorus, amylase, chitinase, xylanase, cellulase, and lignin peroxidase measurements; the second part was stored at 4°C for microbial biomass carbon, nitrogen, and phosphorus, ammonium nitrogen, and nitrate nitrogen analyses; and the third part was preserved in liquid nitrogen at -80°C for subsequent high-throughput microbial sequencing.

### 2.3 Soil physicochemical analyses

Soil pH and electrical conductivity were measured using a multiparameter conductivity/pH tester (MYRON L; Ultrameter II TM, USA). Soil moisture was gravimetrically measured using a 10 g field moist soil sample oven at 105°C for 24 h. SOC content was measured according to the Walkley–Black dichromate oxidation method, and then SOM content was calculated using the following equation: SOM = SOC × 1.724, where 1.724 is the constant coefficient that converts SOC to SOM. Soil microbial biomass nitrogen (MBN) and microbial biomass carbon (MBC) were measured using the chloroform fumigation-extraction method. Total nitrogen (TN) was assessed using a modified micro-Kjeldahl method. Total potassium (TK) were measured with the flame photometry method.Total soil phosphorus and available phosphorus were quantified using a molybdenum-antimony anticolorimetric approach. The activity of soil cellulase (S-CL), amylase (S-AL), basic xylanase (S-BAX), lignin peroxidase (S-Lip), chitinase (CHT) contents were measured using the Cominbio Kits (Suzhou, China).

### 2.4 High-throughput sequencing and analysis

Total genomic DNA of soil was extracted using the CTAB method. The 16S rRNA and ITS genes of the distinct regions were amplified using specific primers (16S V4: 515F–806R, ITS5: ITS3-1737F–ITS2-2043R) (Caporaso et al., 2011). The NEB Next® Ultra™ II DNA Library Prep Kit for Illuminat® (New England Biolabs, USA) was used to generate sequencing libraries. At last, the library was sequenced on an Illumina Nova6000 platform and 250 bp paired-end reads were generated (Guangdong Magigene Biotechnology Co., Ltd. Guangzhou, China). The data were analyzed on the Magigene Cloud Platform (http://cloud.magigene.com). Amplicon Sequence Variants (ASVs) were obtained from the paired-end sequences after quality filtering, denoising and merging using DADA2 in QIIME2 (Callahan et al., 2016). The taxonomy was assigned to each representative sequence using the SILVA v138 (16S rRNA, http://www.arb-silva.de) and Unite v8.0 (ITS rRNA, http://unite.ut.ee) database at a confidence cutoff of 80% (Kõljalg et al., 2013; Quast et al., 2013). Furthermore, FAPROTAX (Louca et al., 2016) and FUNGuild (Nguyen et al., 2016) were used to predict prokaryotic and fungal functions based on ASV taxonomic information, respectively.

### 2.5 Co-occurrence network construction and stability analyses

Network analyses were used to assess the complexity and stability of the microbial community for the blank plots and plots with different recovery years. Spearman’s rank correlation with false discovery rate correction was used to evaluate pairwise associations among ASVs and construct networks for both bacterial and fungal communities. In total, 27 co-occurrence networks were constructed using the “ggClusterNet” R package (Wen et al., 2022). Correlation coefficients > |0.8| with a corresponding of P value < 0.01 were retained. The edges, number of no clusters (NNC), and centralization degree (CD) were calculated using the “network. 2” function in the “ggClusterNet” R package (Wen et al., 2022).

The robustness of each co-occurrence network was then analyzed to evaluate the stability of each network. Robustness was calculated when 60% of random nodes or five targeted module hubs were removed according to Yuan et al (Yuan et al., 2021). Network stability variables, random removal (RR) and targeted removal (TR) of each network, were calculated using the “ggClusterNet” R package (Wen et al., 2022).

### 2.6 Community assembly using null model analysis

Variation partitioning was conducted to determine the relative contribution of spatial versus environmental distances to microbial β-diversity using the “vegan” R package. Null model analyses (999 randomizations) were used to decipher community assembly mechanisms and to provide critical insights into the role of variable selection, homogenous selection, homogenous dispersal, dispersal limitation, and drift in shaping microbial β-diversity. The phylogenetic β-diversity was quantified using beta nearest taxon index (βNTI) by using ‘picante’ package. A value of |βNTI| > 1.96 indicates that the community assembly is governed primarily by deterministic processes, which can be divided into homogeneous selection (βNTI < −1.96, leading to similar community structures in similar environments) and heterogeneous selection (βNTI > 1.96, leading to dissimilar community structures in heterogeneous conditions). While |βNTI| < 1.96, the value suggests that the community compositions are the result of stochastic processes. Additionally, The Bray–Curtis based Raup–Crick (RC_bray_), the deviation between observed Bray–Curtis and the null distribution, was calculated to further partition the stochastic processes. The value of |RC_bray_| > 0.95 represents homogenizing dispersal (RC_bray_ < −0.95) or dispersal limitation (RC_bray_ > 0.95) drives compositional turnover. If |βNTI| < 1.96 and |RC_bray_| < 0.95, this estimates the influence of ‘undominated’ assembly, such as weak selection, weak dispersal, diversification, and/or drift.

### 2.7 Statistical analyses

The LSD test, Student’s t test, Kruskal–Wallis test, and Tukey’s test were performed in SPSS software (SPSS Inc., Chicago, IL, USA). Figures were plotted using the “ggplot2” R packages, ArcGIS and origin2021. The partial least squares path model (PLS-PM) was applied to evaluate the relationships among the restored, soil carbon (soil organic carbon and microbial biomass carbon), soil nitrogen (total nitrogen and microbial biomass nitrogen), bacterial community, fungal community, and enzyme activity. The ‘plspm’ package in R was used to carry out PLS-PM analysis. Bootstrap (1000 iterations) was performed to validate the estimates of path coefficients and the coefficients of determination (R^2^). The Goodness of Fit index was used to evaluate the overall prediction performance of the model.

## 3 Results

### 3.1 Soil physicochemical properties and soil enzyme activity

**Figure 1:**
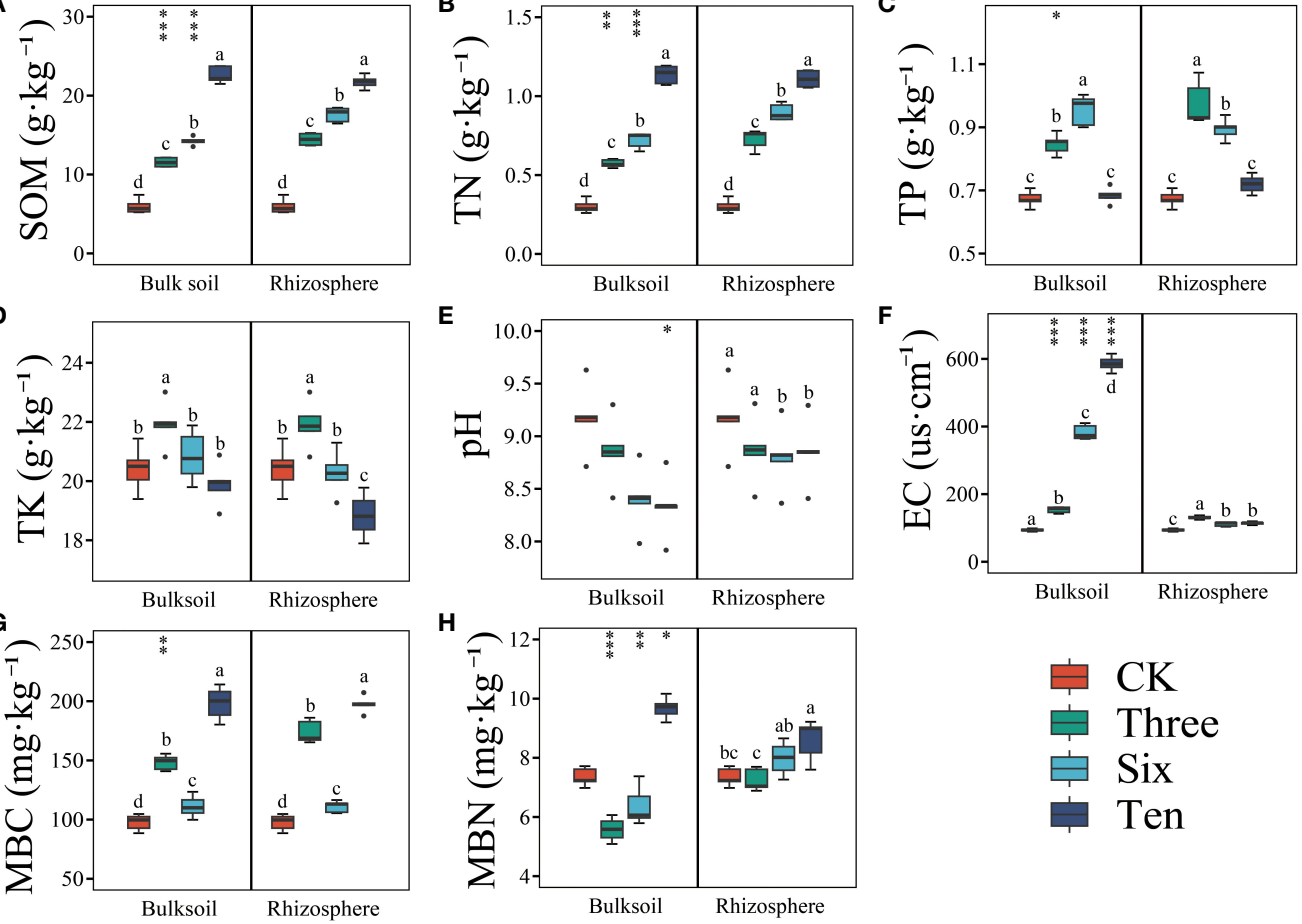
The soil environment variables in the desert land and plantations. Different lowercase letters in each graph indicate a significant difference between different planting years (One-way ANOVA, *P*<0.05). “*”, “**”, and “***” indicate significance level at *P* < 0.05, *P* < 0.01, and *P* < 0.001 difference between rhizosphere and bulk soil of each group, respectively. The method is the Student's t test. SOM **(A)**: soil organic matter; TN **(B)**: soil total nitrogen; TP **(C)**: soil total phosphorus; TK **(D)**: soil total potassium; pH **(E)**; EC **(F)**: soil electrical conductivity; MBC **(G)**: soil microbial biomass carbon; MBN **(H)**: soil microbial biomass nitrogen.

Grapevine cultivation significantly enhanced soil SOM, TN, MBC, and MBN contents (**Figure 1**), and these enhancements intensified over time. For instance, compared to the bare land, the restoration duration led to an increase of 142.05%, 194.43%, and 263.92% in the SOM content of the rhizosphere soil, and 93.44%, 138.56%, and 278.65% in the SOM content of the bulk soil. Similarly, the TN content of the rhizosphere soil increased by 140.99%, 198.31%, and 268.36%, while that of the bulk soil increased by 90.31%, 139.21%, and 277.47% across the respective restoration years. In the third and sixth years, both SOM and TN contents were significantly higher in the rhizosphere than those in the bulk soil, whereas by the 10^th^ year, no significant differences were observed (**Figure 1**).

Moreover, grape cultivation elevated the TP and TK contents in the soil compared to those in bare land (**Figure 1**), however, this was only evident in the 3^rd^ and 6^th^ years, with no significant improvement observed after the 10^th^ year. There were no significant differences between the rhizosphere and bulk soil. Grape cultivation also substantially increased MBC and MBN contents, with a positive correlation with restoration duration. The average MBC and MBN contents in the bare land were 97.61 mg·kg^-1^ and 7.34 mg·kg^-1^, respectively, while by the 10^th^ year, the rhizosphere soil had average MBC and MBN values of 197.39 mg·kg^-1^ and 8.60 mg·kg^-1^, respectively, and the bulk soil had average MBC and MBN values of 198.22 mg·kg^-1^ and 9.68 mg·kg^-1^, respectively. However, the soil pH showed no significant differences (**Figure 1**).

**Figure 2:**
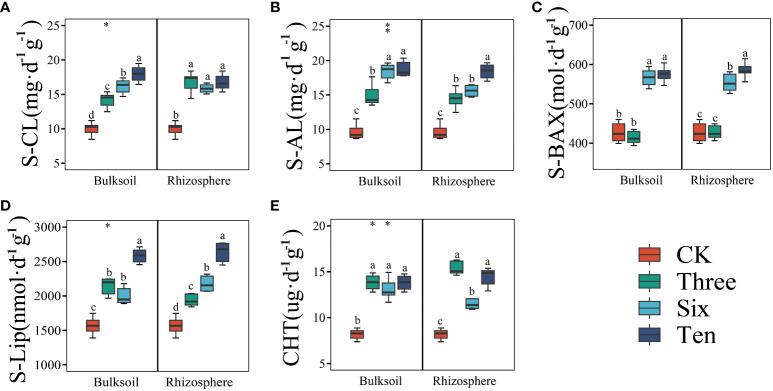
The soil enzyme activity in the desert land and plantations. Different lowercase letters in each graph indicate a significant difference between different planting years (One-way ANOVA, *P*<0.05). “*”, “**”, and “***” indicate significance level at *P* < 0.05, *P* < 0.01, and *P* < 0.001 difference between rhizosphere and bulk soil of each group, respectively. The method is the Student's t test. S-CL **(A)**: soil cellulase; S-AL **(B)**: soil amylase; S-BAX **(C)**: soil alkaline xylanase; S-Lip **(D)**: soil lignin peroxidase; CHT **(E)**: soil chitinase.

In addition, we also measured soil enzyme activity to elucidate the mechanisms of organic carbon transformation in rhizosphere and non-rhizosphere soils. Compared to the bare land, grape cultivation significantly increased the S-CL, S-AL, S-BAX, S-Lip, and CHT contents, showing an upward trend over time (**Figure 2**). However, the effects of this vary within the rhizosphere. For instance, the S-CL content in bulk soil gradually increased with restoration duration; however, no significant differences were observed among the different restoration years in the bulk soil. This was similarly observed in both the rhizosphere and bulk soils of the CHT. In contrast, S-AL, S-BAX, and S-Lip contents exhibited an increasing trend in both the rhizosphere and bulk soils over time.

### 3.2 Microbial diversity and community composition

**Figure 3:**
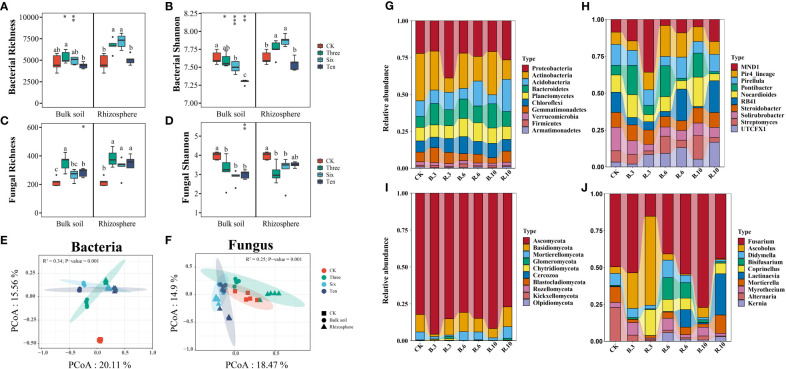
Alpha and beta diversity of the bacterial and fungal communities and community composition. **(A, B)** The bacterial richness and shannon index of the desert land and plantations. **(C, D)** The fungal richness and shannon index of the desert land and plantations. Different lowercase letters in each graph indicate a significant difference between different planting years (One-way ANOVA, *P*<0.05). “*”, “**”, and “***” indicate significance level at *P* < 0.05, *P* < 0.01, and *P* < 0.001 difference between rhizosphere and bulk soil of each group, respectively. The method is the Student's t test. **(E, F)** Principal co-ordinates analysis (Pcoa) and the Anosim test show that plantation altered the bacterial and fungal communities. Relative abundances of soil bacterial and fungal community composition at the phyla **(G, I)** and genes **(H, J)** levels across planting years.

Grapevine cultivation did not significantly increase bacterial richness in the bulk soil but significantly enhanced the richness of the rhizosphere soil (**Figure 3B**), and it increased fungal richness in both soils (**Figure 3D**). The richness of soil microorganisms did not change substantially with restoration (**Figures 3A, C**). Interestingly, in bulk soil, both bacterial and fungal Shannon diversity declined due to grape cultivation, with the former decreasing further with increasing restoration years, whereas the fungal Shannon index showed no significant differences. In rhizosphere soil, compared to the bare land, bacterial α-diversity significantly increased in the 3^rd^ and 6^th^ years, with no difference observed in the 10^th^ year, in contrast to fungi. ANOSIM tests and PCoA revealed significant differences in the soil microbial structure composition between the bare land and grapevine cultivation sites, indicating the formation of distinct clusters of bacteria and fungi after grape cultivation (**Figures 3E, F**). Dominant phyla in microbial communities from bare land and grape cultivation sites were similar at the phylum level but differed at the genus level. Fungi were primarily composed of Ascomycota and Basidiomycota, which accounted for over 80% of the phyla. The predominant bacterial phyla were Proteobacteria, Actinobacteria, Acidobacteria, Bacteroidetes, Planctomycetes, and Chloroflexi (**Figures 3G, J**).

### 3.3 The stability of the soil microbial community

**Figure 4:**
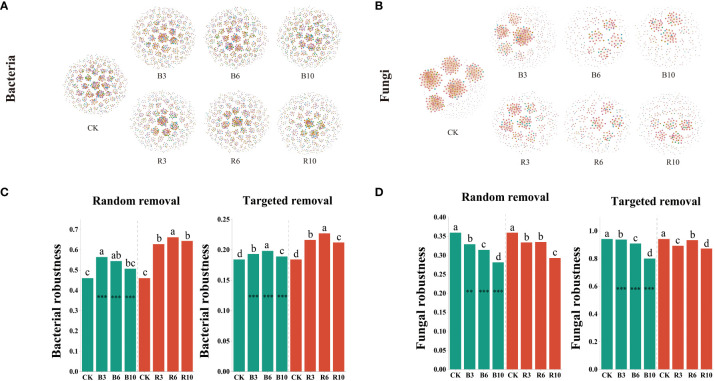
Network stability of bacterial and fungal communities. **(A)** Co-occurrence networks for bacterial community in the desert land and plantations. Rhizosphere soil and bulk soil seasons are represented by the R and the B, respectively. CK, R3, R6, and R10 represented the desert land, three-year, six-year, and ten-year plantations in rhizosphere soil. **(B)** Co-occurrence networks for fungal communities in the desert land and plantations. Rhizosphere soil and bulk soil seasons are represented by the R and the B, respectively. CK, B3, B6, and B10 represented the desert land, three-year, six-year, and ten-year plantations in bulk soil, respectively. **(C, D)** Random removal robustness measures as the proportion of taxa remained with 60% of the taxa randomly removed from each network. Targeted removal robustness measures as the proportion of taxa remained with five module hubs removed from each network. Rhizosphere and bulk soil are shown in red and green, respectively. Each error bar in **(C, D)** corresponds to the standard error of the mean of each column after 100 repetitions of the simulation. Different letters indicate significant differences between desert land, three-year, six-year, and ten-year plantations of each soil using the Kruskal–Wallis test. *** indicates significance level at *P* < 0.001 between growing and non-growing seasons of each group respectively. The method is the Student's t test.

Co-occurrence networks of the bacterial and fungal communities were constructed to assess community stability and complexity (**Figures 4A, B**). On an average, following grape cultivation, the stability of bacterial communities in the rhizosphere soil increased by 5.05% in terms of relative redundancy (RR) and by 5.26% in terms of topological role (TR), whereas in the bulk soil, it increased by 2.56–4.93% in RR and 22.77% in TR. In contrast, the stability of fungal communities decreased by an average of 7.27% in RR and 0.91% in TR in rhizosphere soil, and by an average of 4.53% in RR and 0.48% in TR in bulk soil. Fungal communities exhibited higher thresholds for RR and TR than the bacterial communities (**Figures 4C, D**). Grape cultivation enhanced the stability of bacterial communities in both soils, while decreasing the stability of fungal communities. Additionally, grape cultivation influenced the network complexity metrics of the microbial communities, including the Edge, CD, and NNC. Strikingly, we observed an increase in soil microbial carbon and microbial nitrogen content, along with significant enhancement in fungal richness due to grape cultivation, yet fungal community stability decreased. We hypothesize that this might be due to grape cultivation driving soil microbial community functionality towards a more uniform state. Although fungal biomass increased, the diversity of fungal functions and the coherence among fungal associations were likely reduced, which is supported by the results of fungal Shannon diversity.

### 3.4 Community assembly process

**Figure 5:**
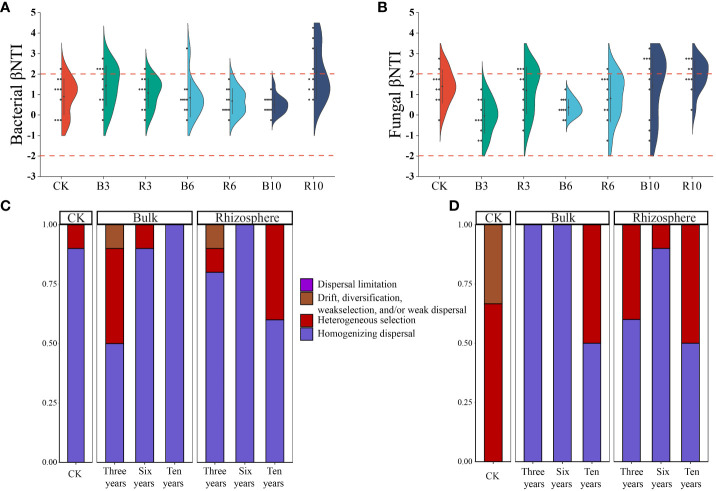
Community assembly process measurements. **(A, B)** were the boxplots of the β-nearest taxon index (βNTI) for all pairs of communities within bacterial and fungal communities in rhizosphere and bulk soils under different grape cultivation years. **(C)** and **(D)** was the relative importance of different ecological processes in rhizosphere soil and bulk soil communities, respectively.

βNTI and RC_Bray_ values were used to examine the effects of different ecological processes on bacterial assembly in the rhizosphere and bulk soils under different grape cultivation years (**Figure 5**). The majority of the βNTI values for both plastisphere and planktonic communities fell between −2 and 2. Simultaneously, the mean values of RC_Bray_ in rhizosphere and bulk soils under different grape cultivation years fell between −0.95 and 0.95. Both values revealed that stochastic processes dominantly mediated microbial assembly in bacterial and fungal community composition in the soils at different times. The stochastic process of homogenizing dispersal caused the assembly of the soil microbial communities, followed by heterogeneous selection. With an increase in grape cultivation time, the assembly of both soil fungal communities gradually shifted from homogenizing dispersal dominance to the coexistence of heterogeneous selection and homogenizing dispersal. Moreover, “undominated” assembly, such as weak selection, weak dispersal, diversification, and/or drift, gradually disappeared in the assembly of the soil fungi communities. For the bulk soil bacterial communities, after 3 years of grape cultivation, the community assembly changed from homogenizing dispersal to the coexistence of that and heterogeneous selection. However, with an increase in cultivation time, the assembly of bacterial communities changed to a homogenizing dispersal. The assembly of the rhizosphere soil bacterial communities gradually changed from homogenizing dispersal dominance to the joint action of heterogeneous selection and homogenizing dispersal.

### 3.5 Function pathways of soil microbes

To compare and analyze the abundance differences of bacterial metabolic functions in rhizosphere and bulk soils under different grape cultivation years, we performed functional statistics in KEGG pathway. In KEGG pathway level 1, Metabolism had an average abundance proportion of about 80%, Genetic Information Processing had an abundance of about 11–12%, Cellular Processes had an abundance proportion of about 4%, and Environmental Information Processing had a function abundance of about 1.9%. In Metabolism function, Carbohydrate metabolism and Amino acid metabolism had the highest abundance. After ten years of grape cultivation, the abundance of pathways such as Carbohydrate metabolism, Amino acid metabolism, Xenobiotics biodegradation and metabolism, Lipid metabolism, Membrane transport, Transport and catabolism in bulk soil was significantly higher than that in rhizosphere soil.

In addition, we also analyzed the fungal functions in rhizosphere and bulk soils under different grape cultivation years, and classified the fungi in rhizosphere and bulk soils into three categories according to their nutritional modes by matching with FUNGuild database: (1) pathotroph; (2) symbiotroph; (3) saprotroph. Among saprotrophic fungi, Undefined Saprotroph had the highest abundance, followed by Dung Saprotroph and Wood Saprotroph (**Supplementary Figure S3**). Among symbiotrophic fungi, Endophyte had the highest abundance. After ten years of grape cultivation, compared with the bulk soil without grape cultivation, the abundance of Endophyte fungi in bulk soil decreased significantly, while the abundance of Wood Saprotroph fungi tended to increase, but the change was not significant. In rhizosphere soil, after ten years of grape cultivation, the abundance of Wood Saprotroph fungi increased significantly. After ten years of long-term grape cultivation, the abundance of Dung Saprotroph fungi in bulk soil was significantly higher than that in rhizosphere soil, while Endophyte was more abundant in rhizosphere soil.

### 3.6 Different roles of the soil microbial community for soil reclamation in rhizosphere and bulk soils

**Figure 6:**
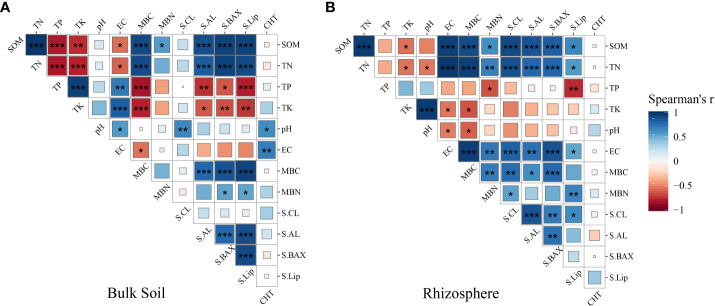
Relationships between soil physicochemical properties, enzyme activities under long-term grape cultivation in bulk soil **(A)** and rhizosphere soil **(B)**. The figure is the Spearman correlation matrix of the physicochemical properties. “*”, “**”, and “***” indicate significance level at *P* < 0.05, *P* < 0.01, and *P* < 0.001. S-CL is soil cellulase, S-AL is soil amylase, S-BAX is soil alkaline xylanase, S-lip is soil lignin peroxidase, CHT is insect chitinase.

To uncover the factors influencing the increase in soil organic carbon in both rhizosphere and bulk soil, we initially investigated the relationship between soil enzymes and physicochemical properties using Spearman correlation coefficients. The results are depicted in **Figure 6**. In bulk soil, Soil Organic Matter (SOM) exhibited significant positive correlations with Total Nitrogen (TN), Microbial Biomass Carbon (MBC), Microbial Biomass Nitrogen (MBN), soil amylase, soil alkaline xylanase, and soil lignin peroxidase, while it showed significant negative correlation with Total Phosphorus (TP), Total Potassium (TK), and Electrical Conductivity (EC). In rhizosphere soil, SOM showed significant positive correlations with TN, EC, MBC, MBN, soil cellulase, soil amylase, soil alkaline xylanase, and soil lignin peroxidase, while it showed negative correlation with TP, TK, and pH. Additionally, we observed that soil enzyme activities mostly exhibited a similar trend in promoting soil organic matter improvement, yet with a more pronounced enhancement evident in the rhizosphere. The key influencing soil enzymes were identified as S-AL, S-BAX, and S-Lip (**Supplementary Figure S4**).

**Figure 7:**
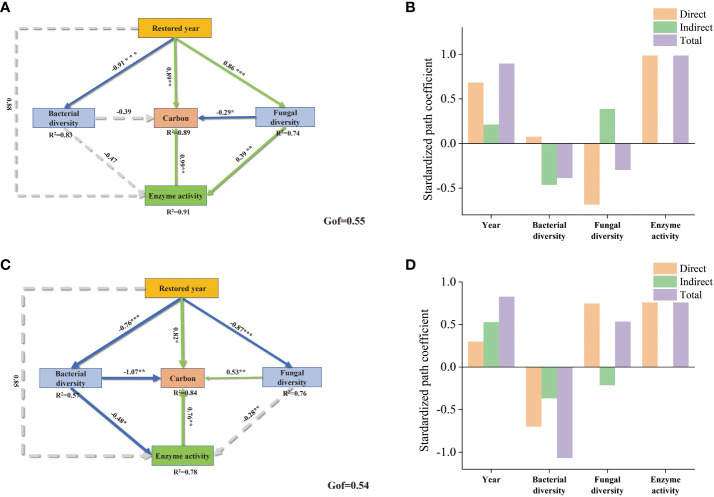
The partial least squares structural equation model (PLS-SEM) for the effects of soil carbon, enzyme activity, bacterial community, fungal community, restored years in bulk soil **(A)** and rhizosphere soil **(C)**; enzyme activity is a latent variable, which is indicated by S-CL, S-AL, S-BAX, S-lip, and CHT. Negative and positive effects are indicated by green and blue line respectively, while non-significant effect is indicated by dashed line. Bar plots show the standard effects on carbon **(B, D)**. “*” indicates significance, “*” for 0.01<*p*<0.05, “**” for 0.001<*p*<0.01, and “***” for *p*<0.001.

We employed Partial Least Squares Path Modeling (PLS-PM) to further comprehend the effects of soil physicochemical properties, enzyme activities, years of grape restoration, and microbial diversity on soil nutrient enhancement (**Figure 7**). In both rhizosphere and bulk soils, enzyme activity was positively correlated with soil carbon content. In the bulk soil, the Goodness of Fit (GOF) of PLS-PM was 0.55, and enzyme activity exhibited the most significant correlation with the improvement of soil carbon content, with a correlation coefficient of 0.99. This was followed by the years of restoration, which had a correlation coefficient of 0.89. The bacterial community and fungi community showed negative correlations with soil carbon content improvement, with correlation coefficients of -0.39 and -0.29 respectively. And the restored year was significantly negatively correlated with the bacterial community, but significantly positively correlated with fungal diversity. For rhizosphere soils, restored year was significantly positively correlated with soil carbon and enzyme activity. Fungi community significantly positively correlated with carbon content in the rhizosphere soil, whereas bacteria showed significantly negative correlation. Restored year had a both significant negative correlation with the bacterial community and fungal community.

## 4 Discussion

### 4.1 Long-term grape cultivation improved soil nutrients

In the context of grape cultivation within desert ecosystems, we observed a significant increase in soil nutrient content, including TN, SOM, TP, and TK over time (Tedersoo et al., 2014). Discrepancies emerged during the 3^rd^ and 6^th^ years, when rhizospheric nutrient levels notably surpassed those in the non-rhizospheric region, which is linked to post-restoration climatic and moisture conditions. Plant root systems play pivotal roles in safeguarding water sources, and the presence of high nutrient concentrations promotes SOM and nitrogen contents, fosters root development, and enables deep water absorption by the soil layers, enhancing drought resistance (Grime, 1977; de Oliveira Garcia et al., 2018). Organic matter aerates the soil, consequently augmenting the water retention capacity (Tang et al., 2021; Wang et al., 2022a). The increase in soil nutrient content may have been influenced by the heightened activity of soil microorganisms, as secretions from the grape root systems can supply soil microorganisms with carbon and energy, stimulating growth (Bardgett et al., 1999; Cheng et al., 2018; Ren et al., 2021). This can accelerate organic matter decomposition within the soil, bolstering the soil nutrient content (Kuzyakov et al., 2007). The absence of variations at the 10-year mark indicates the effects of grape cultivation on soil nutrient enhancement, and this trend was intertwined with the gradual stabilization of soil microbial communities. Microbial communities, adapting to grape cultivation, have achieved a relatively stable status, leading to a more uniform trend in soil nutrient dynamics.

The nutrient cycling mechanisms mediated by microorganisms in the rhizosphere and bulk soils exhibited inconsistencies under long-term grape cultivation. PLS-PM analysis revealed that enzyme activity was beneficial for carbon fixation in sandy bulk soils, whereas the community of fungi and bacteria had a negative correlation with soil carbon in sandy bulk soil. This implies that in the bulk soil, the increase in soil organic matter primarily relies on soil enzymes. This can also be reflected in **Figure 6**, where the Soil Organic Matter (SOM) in the bulk soil showed extremely significant positive correlations with soil amylase, soil alkaline xylanase, and soil lignin peroxidase. Recent research has shown that the topsoil (0-40 cm) is rich in carbohydrates and lignin (38%-50%) (Huang et al., 2023). These soil enzymes can promote the decomposition of recalcitrant organic carbon into small molecules to obtain unstable organic carbon, thereby nourishing the growth of soil microorganisms. Furthermore, in the bulk soil, the abundance of Wood Saprotroph fungi and Dung Saprotroph fungi significantly increased compared to the soils where grapes were not planted and the rhizosphere soils. This suggests that the soil organic matter in the bulk soil primarily originates from the decomposition of plant litter and animal carcasses or feces by soil organisms.

In the rhizosphere soil, in addition to soil enzymes making a significant contribution to root organic matter, the rhizosphere fungal community also significantly contributes to the increase in soil organic carbon. The interaction between border cells of plant roots and microorganisms can release flavonoids that attract rhizosphere microorganisms, inducing the differentiation of rhizosphere fungal hyphae (Sasse et al., 2018). Several studies have shown that fungal necromass is the main component of Soil Organic Matter (SOM) (Wang et al., 2019; Sae-Tun et al., 2022). In rhizospheric soils, fungi aid in storing Soil Organic Carbon (SOC), and extensive fungal mycelia can form stable soil aggregates (Sanaullah et al., 2020). Furthermore, the abundance of wood saprotrophic fungi and endophyte fungi in the rhizospheric soil increased under long-term grape cultivation. Endophyte fungi can activate plant root nutrients and transfer plant-fixed carbon to the soil (Bell et al., 2022; Duan et al., 2023). Therefore, we speculate that the SOC in the grape rhizospheric soil originates from the fungal decomposition of grape roots and the small molecule carbon sources fixed by plant roots.

### 4.2 Effects of long-term grape cultivation on soil microbial communities

Following the initial planting of grapes, there was an increase in the richness of both bacteria and fungi, while their evenness decreased. Effective soil nutrient enhancement, Soil Organic Carbon (SOC) modulation, and nutrient availability through grape cultivation have shaped the composition of microbial communities (Cao et al., 2008; Pingree and DeLuca, 2018). In bulk soil, both bacterial and fungal Shannon indices declined due to grape cultivation, with bacterial Shannon diversity decreasing further with increasing restoration years, while fungal Shannon diversity showed no significant differences among restoration years. Gradual water limitations can cause fluctuations in microbial network stability, with fungi being less responsive to nutrient changes (Deng et al., 2020). Furthermore, both bacterial and fungal network stabilities decreased with increasing recovery time. Fungal communities exhibited higher RR and TR thresholds than bacterial communities, as grape cultivation in desert areas intensifies soil aridity, thus diminishing the stability of soil microbial communities (de Vries et al., 2018; Shi et al., 2020; Hernandez et al., 2021). Our study suggests that different taxa drive changes in microbial symbiotic networks and post-cultivation stability; therefore, the increased stability of the bacterial symbiotic network following grape cultivation may result from elevated soil carbon and nitrogen levels (de Vries et al., 2018). Microbial symbiosis analyses revealed an increase in the soil microbial bacterial network stability and a decrease in the fungal network stability following grape cultivation (Li et al., 2021; Hu et al., 2022). Under monoculture planting, although soil nutrients are effectively enhanced, bacterial and fungal ecological niches are reduced, intensifying competition between them and consequently reducing network stability (Blagodatskaya and Kuzyakov, 2008; Hernandez et al., 2021). Additionally, our data indicate that with increasing years of cultivation, the EC content in the bulk soil continues to rise. Due to the arid climatic conditions of the study site characterized by year-round drought, grape cultivation further reduces soil moisture, leading to an increase in soil EC, which directly affects microbial growth and metabolic activities (Kieft et al., 1987; Evans and Wallenstein, 2014; Na et al., 2019). Under dry conditions and high-salt soils, limitations in microbial nutrient availability could be intensified (Fuchslueger et al., 2014; Meisser et al., 2019). Therefore, we speculate that in the bulk soil, long-term grape cultivation leads to a reduction in ecological niches and an increase in salinity, resulting in a decrease in soil microbial diversity. Furthermore, due to the differences in the physicochemical properties of rhizospheric and bulk soils, particularly the secretion of plant root substances such as flavonoids and phenols, microbial community assembly could be profoundly influenced (Phillips and Fahey, 2006; Nuccio et al., 2020). We also found that fungi possess greater resistance to disturbances than bacteria. Compared to bacteria, fungi possess sufficient capacity to tolerate harsh environments, which could be attributed to physiological traits, such as reproductive modes (spore production, aerial spore dissemination, and active dispersal via aerial hyphae) (Roper et al., 2010; Barnard et al., 2013; Wu et al., 2021).

## 5 Conclusion

**Figure 8:**
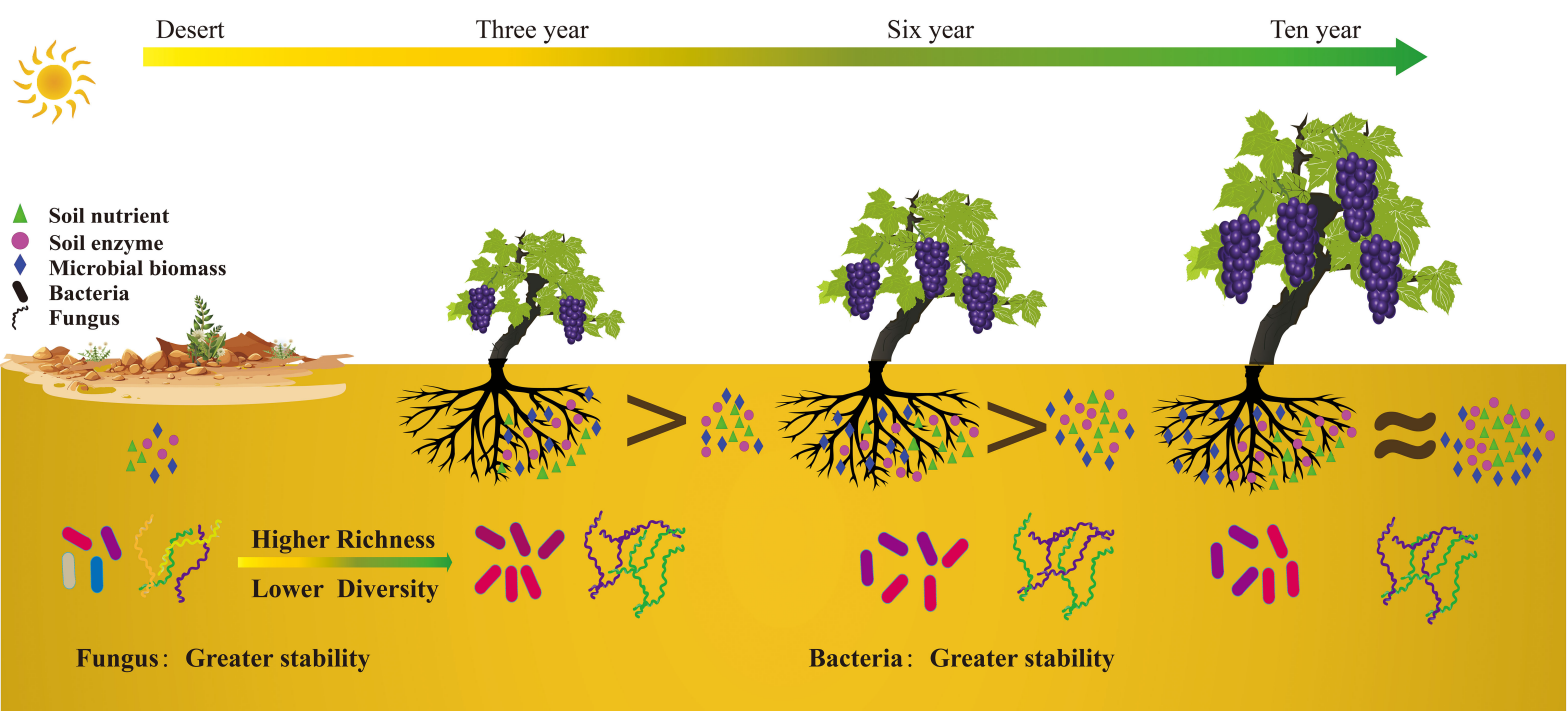
Schematic illustration showing how long-term cultivation of wine grapes enhances soil organic carbon content in desert ecosystems.

In this study, we investigated the effects of extended grape cultivation on the nutrient composition of soil and microbial communities in both rhizosphere and bulk soils (**Figure 8**). Our results show that after six years grape cultivation, the soil organic matter (SOM) significantly increased from 7 g/kg prior to cultivation to approximately 22 g/kg, thereby improving soil quality. Grape cultivation also alters the structure and diversity of the soil microbial community in both soil types. In bulk soil, long-term grape cultivation results in a decrease in ecological niches and an increase in salinity, leading to a reduction in soil microbial diversity. Moreover, we found that soil enzymes and microorganisms play crucial roles in enhancing SOM content. In bulk soil, soil enzymes are the main contributors to SOM enhancement, decomposing recalcitrant organic carbon into small-molecule organic carbon. In rhizosphere soil, microorganisms synthesize organic carbon from plant residues or root exudates and increase the soil organic carbon content by decomposing plant fine roots and producing fungal mycelia. This study provides new insights into the mechanisms of soil nutrient enhancement during extended grape cultivation.

## Data availability statement

The original contributions presented in the study are publicly available. This data can be found here: PRJNA1073022, PRJNA1073288.

## Author contributions

ZW: Writing – review & editing, Writing – original draft, Software, Methodology, Investigation, Formal analysis, Data curation, Conceptualization. WL: Writing – review & editing, Software, Methodology, Investigation, Formal analysis, Data curation. YjW: Writing – review & editing, Methodology, Investigation, Data curation. TM: Writing – review & editing, Methodology, Investigation, Data curation. XW: Writing – review & editing, Methodology, Investigation, Data curation. YL: Writing – review & editing. YqW: Writing – review & editing, Validation, Supervision, Resources, Project administration, Methodology, Investigation, Funding acquisition, Data curation, Conceptualization.

## Supplementary material

Supplementary Figure S1 The study site and the distribution of three vineyards and desert land within this region.

Supplementary Figure S2 The schematic diagram of the experimental design, rainfall and temperature data during the experiment.

Supplementary Figure S3 Function pathways of soil microbes in rhizosphere and bulk soils. **(A)** A bar chart for fungal functional groups (guilds) inferred by FUNGuild in the bulk and rhizosphere soil under different grape cultivation years. Relative abundances of 12 fungal functional groups (guilds) inferred by FUNGuild that were significantly different in **(B)** the bulk soil after ten years of grape cultivation compared with the soil without grape cultivation, **(C)** the rhizosphere soil after ten years of grape cultivation compared with the soil without grape cultivation, and **(D)** the rhizosphere and bulk soil after ten years of grape cultivation, according to a t-test and Wilcoxon rank sum test. “*”, “**”, and “***” indicate significance level at *P* < 0.05, *P* < 0.01, and *P* < 0.001.

Supplementary Figure S4 General linear model of the relationship between S-CL, S-AL, S-BAX, S-Lip and CHT and soil organic matter on rhizosphere and bulk soil.
