# Supplementary figures and images for: Microbiological impact of long-term wine grape cultivation on soil organic carbon in desert ecosystems: a study on rhizosphere and bulk sandy soils
Source: Front Plant Sci. 2024 Mar 7;15:1362149. doi: 10.3389/fpls.2024.1362149 (PMC10955057; doi:10.3389/fpls.2024.1362149)

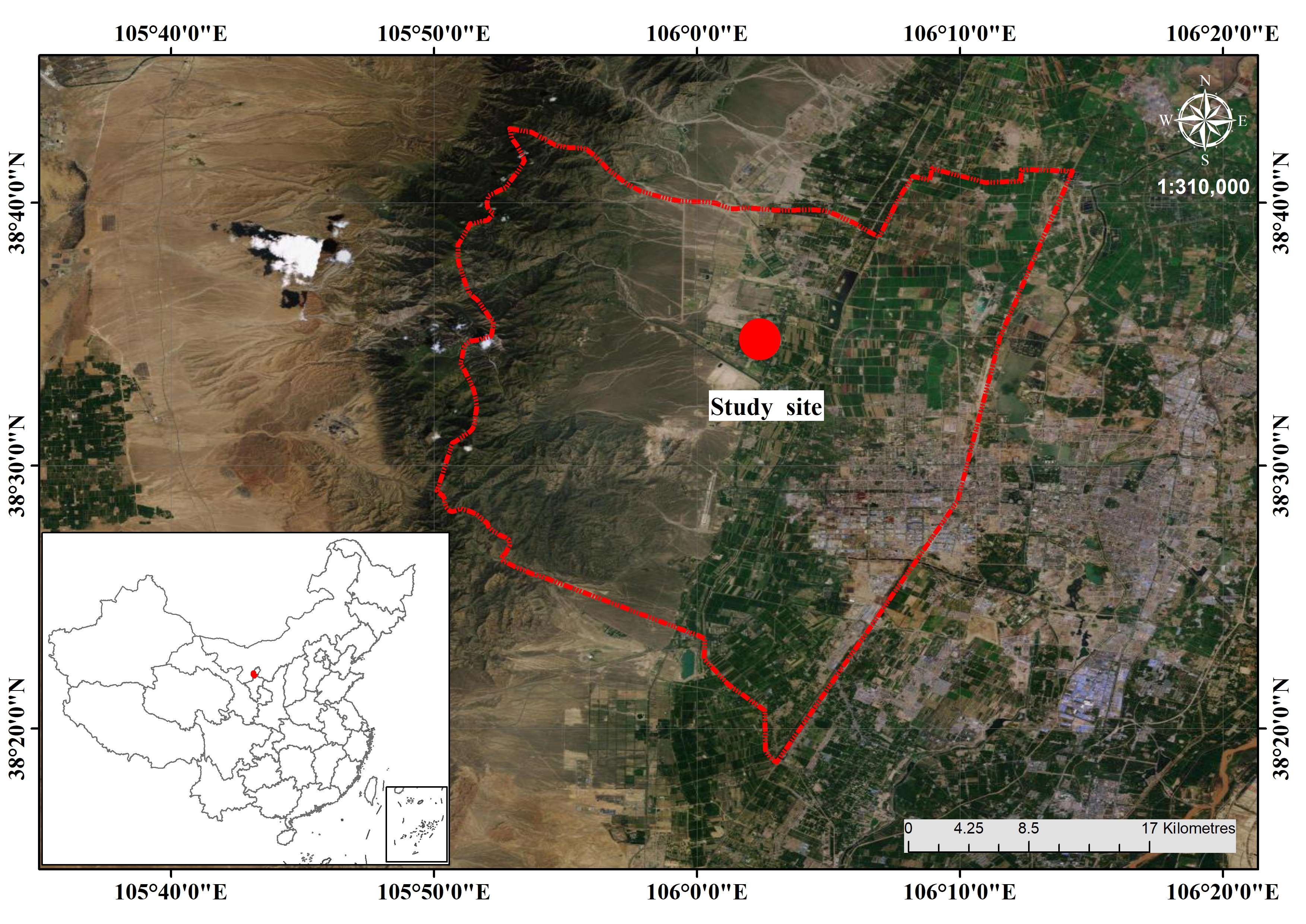

Supplement: Supplementary Figure S1 — The study site and the distribution of three vineyards and desert land within this region. [file Image_1.jpeg]

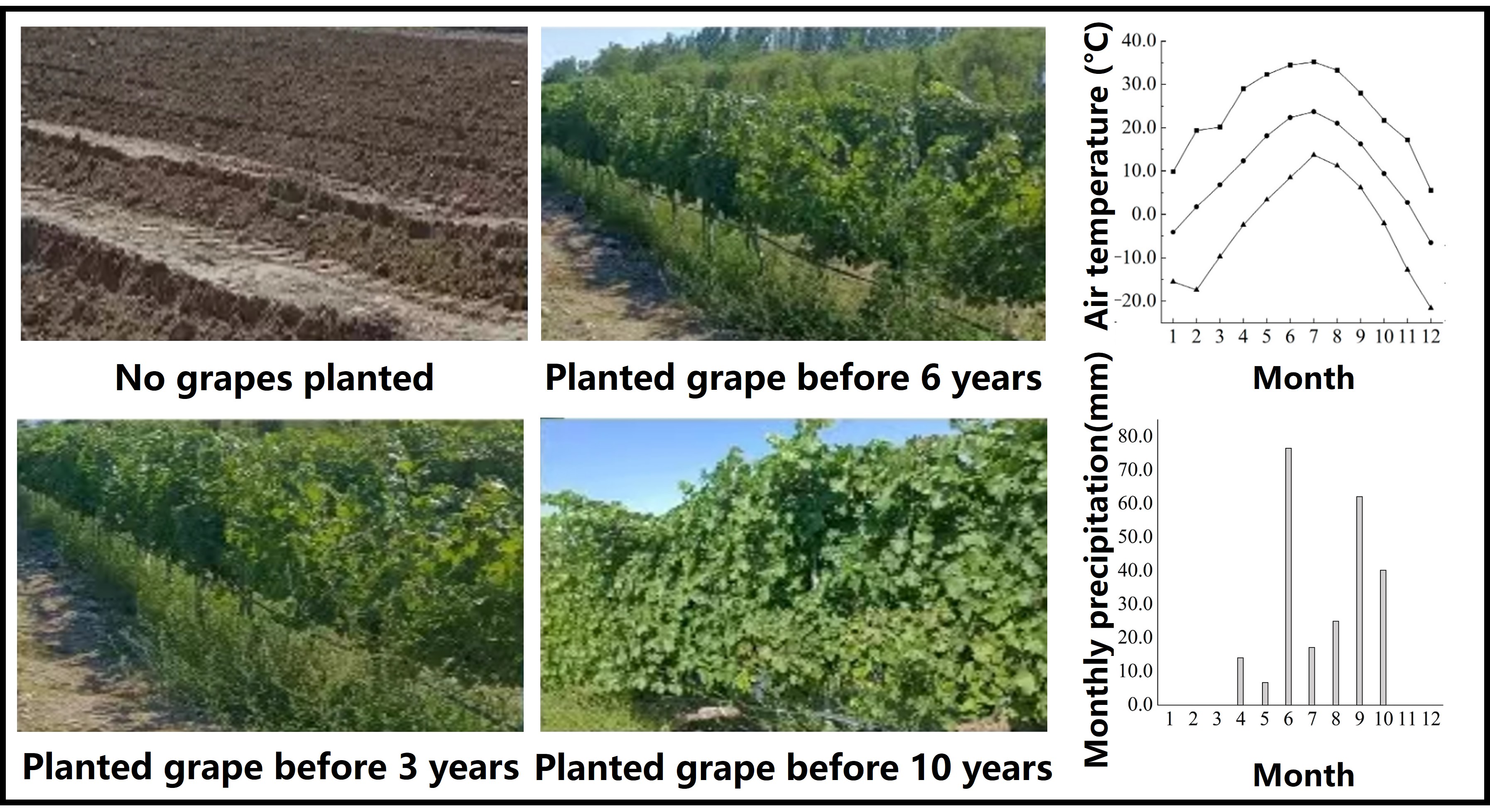

Supplement: Supplementary Figure S2 — The schematic diagram of the experimental design, rainfall and temperature data during the experiment. [file Image_2.jpeg]

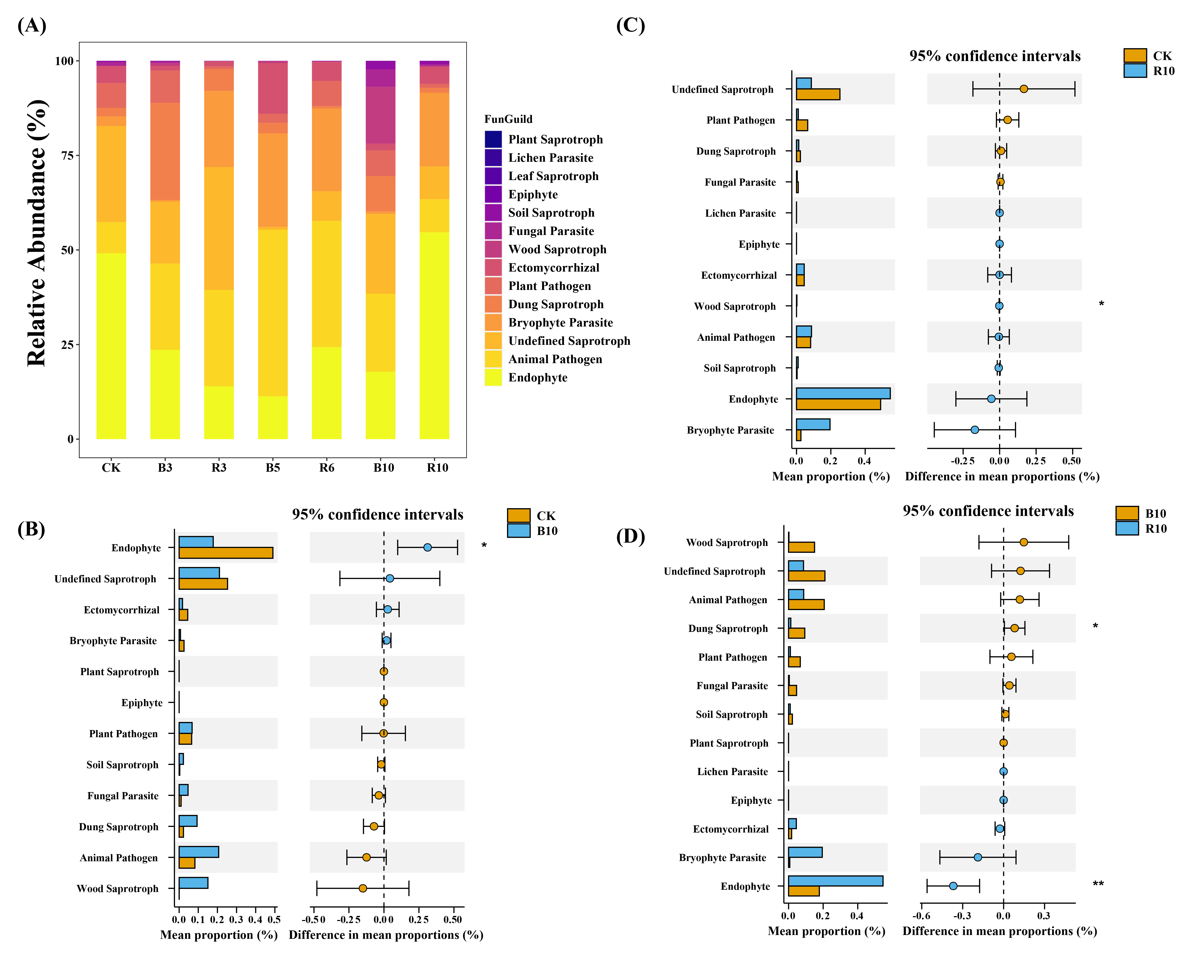

Supplement: Supplementary Figure S3 — Function pathways of soil microbes in rhizosphere and bulk soils. (A) A bar chart for fungal functional groups (guilds) inferred by FUNGuild in the bulk and rhizosphere soil under different grape cultivation years. Relative abundances of 12 fungal functional groups (guilds) inferred by FUNGuild that were significantly different in (B) the bulk soil after ten years of grape cultivation compared with the soil without grape cultivation, (C) the rhizosphere soil after ten years of grape cultivation compared with the soil without grape cultivation, and (D) the rhizosphere and bulk soil after ten years of grape cultivation, according to a t-test and Wilcoxon rank sum test. “*”, “**”, and “***” indicate significance level at P < 0.05, P < 0.01, and P < 0.001. [file Image_3.jpeg]

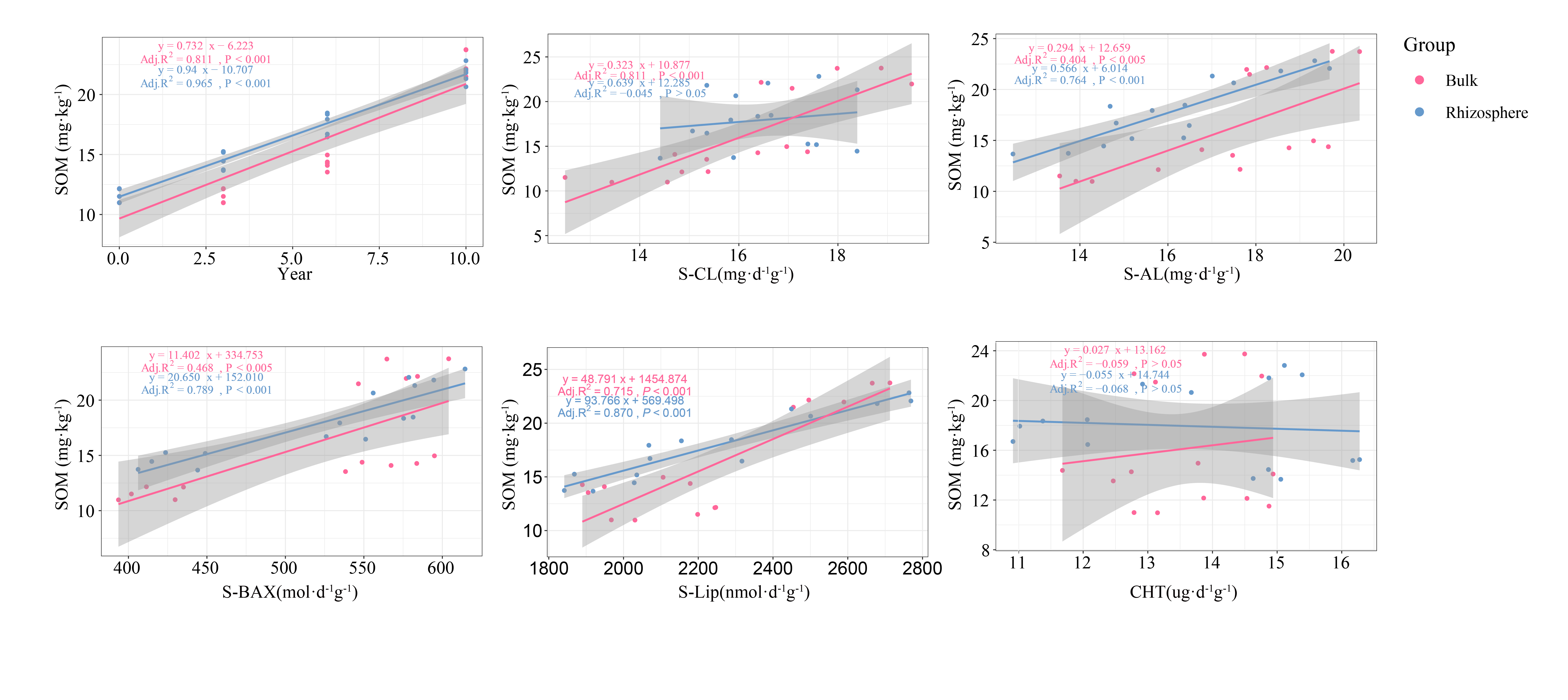

Supplement: Supplementary Figure S4 — General linear model of the relationship between S-CL, S-AL, S-BAX, S-Lip and CHT and soil organic matter on rhizosphere and bulk soil. [file Image_4.jpeg]
